# The role of the microbial-immune-bone axis in bone tumor development: mechanistic integration, systems modeling, and intervention prospects

**DOI:** 10.3389/fcimb.2026.1762046

**Published:** 2026-02-04

**Authors:** Yan Guo, Xue Wang, Yaokun Wu, Yongzhong Li, Xueyan Wei

**Affiliations:** 1 Department of Oncology, Mianyang Fulin Hospital, Mianyang Fulin Hospital Co., Ltd, Mianyang, China; 2 Department of Radiology, Mianyang Fulin Hospital, Mianyang Fulin Hospital Co., Ltd, Mianyang, China; 3 Department of Oncology, Luxian People’s Hospital, Luzhou, China

**Keywords:** bone tumors, microbial metabolites, microbiome-targeted interventions, microbiota–immune–bone axis (MIB), osteoimmunology

## Abstract

The emergence and development of bone tumors stem from a combination of intrinsic genetic alterations in tumor cells, remodeling of the bone marrow microenvironment, and shifts in the host’s systemic immune-metabolic state. In recent years, gut microorganisms have been shown not only to influence bone mass regulation and conditions involving disrupted bone homeostasis, such as osteoporosis, but also to substantially affect the formation of primary bone tumors and metastatic lesions by modulating immune cell differentiation, inflammatory activity, and the coupling of bone remodeling. Focusing on the “Microbiota–Immune–Bone axis” (MIB), a growing body of fundamental and translational research indicates that alterations in gut microbial composition and function can reshape metabolite profiles—including short-chain fatty acids, bile acids, indole derivatives—and pathogen-associated molecular patterns (PAMPs). These signals act on the intestinal barrier and bone marrow immunity through G-protein–coupled receptors, nuclear receptors, and pattern-recognition receptors, thereby shifting the balance between bone resorption and formation and modifying the immune characteristics of the bone microenvironment, ultimately facilitating bone tumor cell colonization, proliferation, and immune escape. This review takes the MIB axis as its central framework to integrate the major pathways through which gut microbes and their metabolites regulate intestinal and myeloid immunity, bone remodeling, and bone tumor biology, to construct a systems-level model of tumor initiation and progression, to identify druggable signaling nodes, and to assess the potential and challenges of microbiota-modulating approaches—including antibiotics, probiotics, dietary strategies, and fecal microbiota transplantation—in preventing and treating bone tumors, thereby offering a theoretical foundation for developing integrated interventions targeting the gut microbiota and the MIB axis.

## Introduction

Bone tumors, particularly malignant primary bone tumors and bone metastases from solid tumors, are major causes of disability and death in patients (Coleman, 2006; Hauben and Hogendoorn, 2015; Macedo et al., 2017; Huang et al., 2020; Bădilă et al., 2021). Traditional research has largely concentrated on the mutational landscape of tumor cells, the RANKL-driven overactivation of osteoclasts, and biological events within the local bone marrow microenvironment, including angiogenesis, inflammatory responses, and immunosuppressive processes (Xing et al., 2005; Ming et al., 2020; Casimiro et al., 2021; Li et al., 2022). Although molecular targeted therapies and immunotherapies have yielded significant progress, genuinely breakthrough mechanisms capable of managing advanced bone metastases and curbing the high rate of skeletal-related complications are still absent. This situation underscores that bone tumors should not be considered merely “localized diseases,” but must be interpreted within the context of the body’s systemic immune and metabolic networks (McDade, 2003; McDade, 2005; Kepp et al., 2020; Cugini et al., 2021).

The gut microbiota, often described as a “neglected organ,” is intimately linked to the host’s immune maturation, energy metabolism, endocrine function, and neuroendocrine regulation (Shanahan et al., 2017). The concept of the “gut–bone axis” was originally introduced to describe the connections between intestinal absorption, calcium–phosphorus metabolism, and bone mass changes (Tu et al., 2021; de Sire et al., 2022; He and Chen, 2022; Zhang et al., 2023). As our understanding of gut microbiota–immune interactions has advanced, the field has moved beyond classical nutrient-absorption paradigms toward recognizing the gut microbiota as an active regulator of systemic immune tone. This conceptual shift has laid the groundwork for linking intestinal microbial signals to immune-mediated regulation of bone remodeling (D’Amelio and Sassi, 2018; Tsukasaki and Takayanagi, 2019; Singh et al., 2023).

Consistent with this conceptual framework, experimental and translational studies have demonstrated that gut microbiota–driven immune modulation has measurable effects on bone remodeling and therapeutic responsiveness. These effects are strongly influenced by metabolites like short-chain fatty acids, bile acids, and indole derivatives, as well as receptor-mediated signaling pathways activated by these metabolites. Additionally, the gut microbiota has been found to impact the efficacy and toxicity of immune checkpoint inhibitors, radiotherapy, chemotherapy, and certain targeted therapies (Alexander et al., 2017; Gori et al., 2019; Fernandes et al., 2022; Chrysostomou et al., 2023; Simpson et al., 2023), implying that the host microbial status not only shapes the biological basis of bone tissue, but may also, by modulating antitumor immune responses and bone remodeling, affect the natural course of bone tumors. However, current research on the connection between the gut microbiota and bone tumors remains fragmented. Some studies are restricted to describing changes in microbial community structure, while others focus on a single metabolite or signaling pathway, or examine specific immune cell populations. There is a lack of a comprehensive systems model that integrates the entire “microbiota–metabolites–immunity–bone remodeling–tumor progression” axis.

Focusing on the role of the microbiota–immune–bone (MIB) axis in the initiation and progression of bone tumors, this review summarizes current evidence on how gut microbes and their metabolites (Tu et al., 2021; Chen et al., 2022), via intestinal mucosal immunity and bone marrow myeloid immunity, regulate the RANKL/OPG axis, Wnt signaling, and related bone remodeling pathways, thereby influencing tumor initiation, progression, and formation of the bone metastatic niche. It further consolidates these seemingly unrelated mechanisms into a visual systems model that emphasizes key nodes and signaling pathways, offering a structured conceptual framework for experimental and translational research. The model also examines, in light of recent advancements in antibiotic use, probiotics and synbiotics, dietary modulation, and fecal microbiota transplantation, the feasibility, risks, and unresolved challenges of targeting the MIB axis for bone tumor intervention.

Importantly, this review differs from existing literature in several key aspects. Previous reviews have largely focused on isolated components of the gut–bone axis, such as individual microbial taxa, specific metabolites, or single immune-cell populations, often within the context of metabolic bone diseases. In contrast, the present work centers explicitly on bone tumors and bone metastases and introduces the microbiota–immune–bone (MIB) axis as an integrated, systems-level framework.

By incorporating immune reprogramming, bone remodeling dynamics, and the unique anatomical features of the bone marrow niche, we move beyond descriptive associations to propose a mechanistic and spatially informed model of tumor progression. Furthermore, this review emphasizes actionable intervention anchors across microbial, immune, and skeletal compartments, providing a conceptual roadmap for future experimental design and translational strategies.

**Figure 1:**
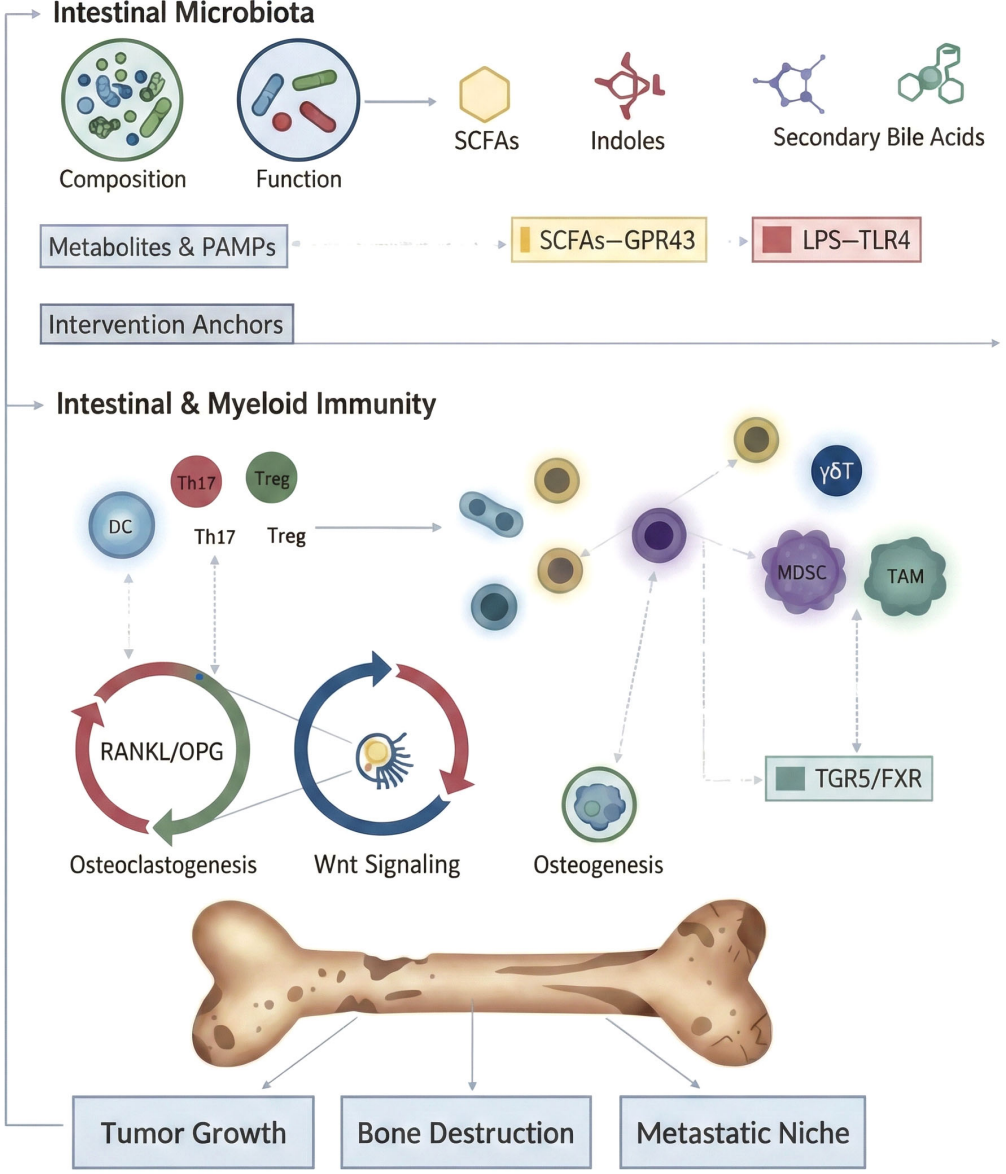
Schematic overview of how alterations in intestinal microbiota–derived metabolites and PAMPs modulate intestinal and myeloid immunity (via pathways such as SCFAs–GPR43 and LPS–TLR4) to influence osteoclastogenesis and osteogenesis, thereby promoting tumor growth, bone destruction and the formation of a pro-metastatic bone niche.

By synthesizing existing research, we develop an integrative systems model that starts with gut microbiota dysbiosis and, through metabolite- and PAMP-mediated modulation of intestinal and myeloid immunity, ultimately leads to an imbalance in bone remodeling and the progression of bone tumors, as illustrated in **Figure 1**.

This diagram offers a comprehensive overview of how the intestinal microbiota influences bone tumor progression via the microbiota–immunity–bone (MIB) axis. Starting with “Intestinal Microbiota” at the top, it highlights how changes in microbial composition and function lead to the production of various metabolites, such as short-chain fatty acids (SCFAs), indole derivatives, and secondary bile acids, along with pathogen-associated molecular patterns (PAMPs) like lipopolysaccharide (LPS). These microbial signals are detected by the host through key pathways, including SCFA–GPR43 and LPS–TLR4. This section is labeled “Metabolites & PAMPs” and marked as potential “Intervention Anchors”.

The middle layer, “Intestinal & Myeloid Immunity,” illustrates how gut-derived cues first regulate dendritic cells (DCs) and the T-cell subsets they induce, such as Th17 and Treg cells, thereby reshaping adaptive immune responses. These immune shifts subsequently influence γδT cells, myeloid-derived suppressor cells (MDSCs), and tumor-associated macrophages (TAMs), representing alterations in both myeloid and innate immunity (Ugel et al., 2015; Haist et al., 2021; Veglia et al., 2021).Several downstream effects are mediated through bile-acid receptors, including TGR5 and FXR. The lower portion of the diagram connects osteoclastogenesis and osteogenesis through the RANKL/OPG axis and the Wnt-signaling module, highlighting that immune-cell alterations and their signaling networks disrupt the dynamic balance of bone remodeling. This imbalance manifests as enhanced bone resorption or impaired bone formation, ultimately contributing to progressive bone destruction and structural remodeling within the bone microenvironment (Parfitt, 1987; Raisz, 1999; Yang and Liu, 2021).At the bottom, the skeletal illustration and arrows pointing toward “Tumor Growth,” “Bone Destruction,” and “Metastatic Niche” summarize the functional consequences of dysregulation along the microbiota–immune–bone axis. Microbial communities and their metabolites orchestrate multilayered modulation of intestinal and bone-marrow immune networks, creating an inflammatory and immunosuppressive bone microenvironment that favors tumor cell proliferation and immune evasion (Patras et al., 2023). These changes stimulate osteoclastic activity, promote bone loss, and create a metastatic bone niche that facilitates the seeding and survival of circulating tumor cells. In summary, the diagram implies that targeting the gut microbiota, key metabolites, and their receptor-mediated pathways could offer upstream control to reprogram immune and bone-remodeling processes, providing a holistic strategy to inhibit bone tumor growth, bone degradation, and metastatic niche formation.

The initiation and malignant progression of bone tumors are tightly associated with immune dysregulation, chronic inflammation, excessive osteoclast activation, suppression of osteoblast function, and remodeling of the bone-marrow microenvironment. Importantly, many of these processes are profoundly governed by gut microbial metabolites. The intestinal microbiota—including abundant anaerobes, lactic acid bacteria, Bacteroides, and Clostridia—ferments dietary fibers, proteins, and lipids to generate bioactive molecules such as SCFAs, indole compounds, secondary bile acids, TMAO, and LPS (Zhang and Davies, 2016; Vernocchi et al., 2020; Debnath et al., 2021). These metabolites enter the bloodstream and interact with receptors—such as GPR41/43, AhR, FXR, TGR5, and TLR4—on immune cells, osteoblasts, osteoclasts, and bone-marrow stromal cells. In doing so, they activate or suppress specific signaling pathways that influence immune responses, inflammation, bone remodeling, and the survival and proliferation of tumor cells within bone.

A critical and often underappreciated question is why gut microbiota–derived metabolites and immune signals exert disproportionately strong effects on bone tumors and bone metastases, rather than on other highly perfused organs such as the liver or lung. A key explanation lies in the unique anatomical and immunological features of the bone marrow microenvironment. Unlike continuous capillary systems, the bone marrow is characterized by a sinusoidal vascular network with high permeability, low shear stress, and discontinuous endothelial junctions, which facilitates efficient exposure of resident immune cells and stromal components to circulating metabolites and pathogen-associated molecular patterns. Moreover, the bone marrow harbors a dense population of myeloid progenitors, osteoclast precursors, and immune-regulatory cells that are exquisitely sensitive to systemic inflammatory and metabolic cues. Continuous bone remodeling further amplifies this sensitivity by generating localized inflammatory gradients, growth factors, and chemokines that collectively create a permissive niche for tumor cell homing and survival. Together, these anatomical and functional features render the bone marrow a preferential target organ in which microbiota-driven immune–metabolic perturbations are translated into tumor-promoting signals.

**Figure 2:**
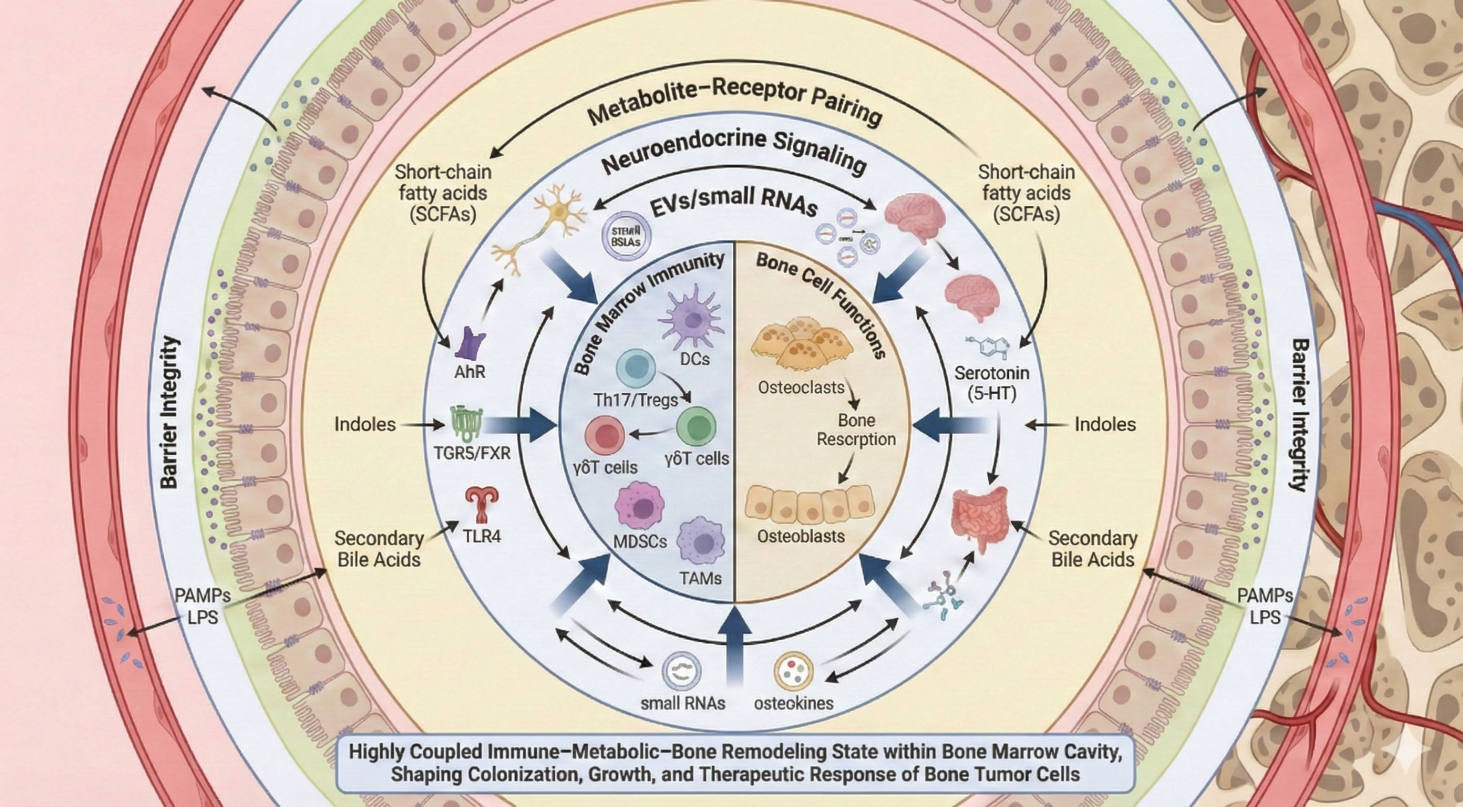
The microbiota–immune–bone (MIB) axis operates through four interconnected communication channels—barrier integrity, metabolism–receptor pairing, neuroendocrine signaling, and EVs/small RNAs—by which gut-derived signals converge on bone marrow immunity and bone cell function to shape the bone microenvironment relevant to tumor progression.

To explore how the MIB axis initiates bone tumorigenesis, we start with microbial metabolites. SCFAs (such as acetate, propionate, and butyrate) facilitate Treg differentiation via GPR43 activation. Tregs are critical immunoregulatory cells in the bone microenvironment that restrain excessive Th17 activity, reduce inflammatory burden, and maintain a stable immune milieu conducive to limiting bone resorption and supporting mineralization (Zhu et al., 2020; Yang and Liu, 2021). Under normal conditions, this maintains a protective balance for bone integrity. However, in the case of microbial dysbiosis, SCFAs production decreases, Treg numbers fall, and Th17 cells proliferate. This shift raises levels of inflammatory mediators like IL-17 and TNF-α, which not only enhance osteoclast differentiation and activation but also create an “inflammatory microenvironment” that supports tumor cell engraftment in bone, establishing a pre-metastatic niche. It is crucial to note that the cascade shown in **Figure 1** does not follow a single signaling pathway; instead, it is driven by a multilayered, multimodal flow of information. From a systems biology viewpoint, the microbiota–immune–bone (MIB) axis involves at least four interconnected communication channels: barrier integrity, metabolite–receptor interactions, neuroendocrine regulation, and extracellular vesicle/small RNA-mediated signaling. Together, these pathways shape the immune environment of the bone marrow and the functional state of bone cells. To capture this, we developed a “four-channel communication” model to integrate and visualize the key information flows within the MIB axis (**Figure 2**).

**Figure 2** concisely outlines a concentric “four-pathway communication” model of the MIB axis linking the gut microbiota with bone marrow immunity and bone cell function. At the center are two core modules: Bone Marrow Immunity and Bone Cell Function. The former comprises major effector populations, including dendritic cells (DCs), Th17/Treg cells, γδT cells, myeloid-derived suppressor cells (MDSCs), and tumor-associated macrophages (TAMs). The latter reflects the outcomes of bone remodeling, represented by osteoclast-mediated bone resorption and osteoblast functional states. Converging arrows indicate that peripheral signals ultimately feed into these two modules, where they are integrated.

The concentric rings radiating outward depict four major gut–bone communication pathways. The outermost pathway, Barrier Integrity, preserves intestinal barrier function through tight junctions, the mucus layer, and antimicrobial peptides, thereby restricting translocation of lipopolysaccharides (LPS) and other pathogen-associated molecular patterns (PAMPs) into circulation and reducing downstream TLR4-driven inflammatory activation within the bone marrow. The Metabolism–Receptor Pairing pathway highlights the coupling of microbiota-derived metabolites—including short-chain fatty acids (SCFAs), indole derivatives, and secondary bile acids—with host receptors (such as AhR, TGR5/FXR, and TLR4). Through these ligand–receptor interactions, microbial signals modulate immune polarization and intracellular signaling in osteocytes, ultimately reshaping the bone microenvironment. The Neuroendocrine Signaling pathway, critically represented by mediators such as serotonin (5-HT), links intricate gut–brain–bone neuroendocrine cues to bone remodeling, immunological regulation, and potentially tumor development. Notably, a significant portion of the body’s serotonin is produced by enterochromaffin cells in the gut, with its synthesis and bioavailability profoundly influenced by the gut microbiota. This intestinal serotonergic system intricately cross talks with host pattern recognition receptors (PRRs), playing a pivotal role in shaping both local intestinal and systemic immune responses (Layunta et al., 2021). By modulating immune cell activity—including the balance of pro-inflammatory and anti-inflammatory cytokines—and directly influencing osteocyte function, 5-HT not only impacts bone remodeling dynamics but also contributes to the establishment of an immune microenvironment that can either suppress or promote tumor cell colonization and progression within the bone. This captures how nutritional status, emotional state, and stress, acting via neuroendocrine pathways, collectively influence bone marrow immunity and osteocyte function, thereby impacting the MIB axis. The innermost ring, EVs/small RNAs, emphasizes extracellular vesicles and their cargo (including small RNAs and osteokines) as vehicles for long-range, bidirectional communication between gut and bone, directly reprogramming gene-expression profiles in immune cells and osteocytes.

In summary, the four pathways depicted in the diagram are not independent; rather, they are tightly interconnected across temporal and spatial scales. Acting in concert, they shape the immune–metabolic–bone remodeling landscape within the bone marrow cavity, creating a dynamic and adaptable ecosystem that influences bone tumor cell colonization, outgrowth, and therapeutic responsiveness.

**Figure 3:**
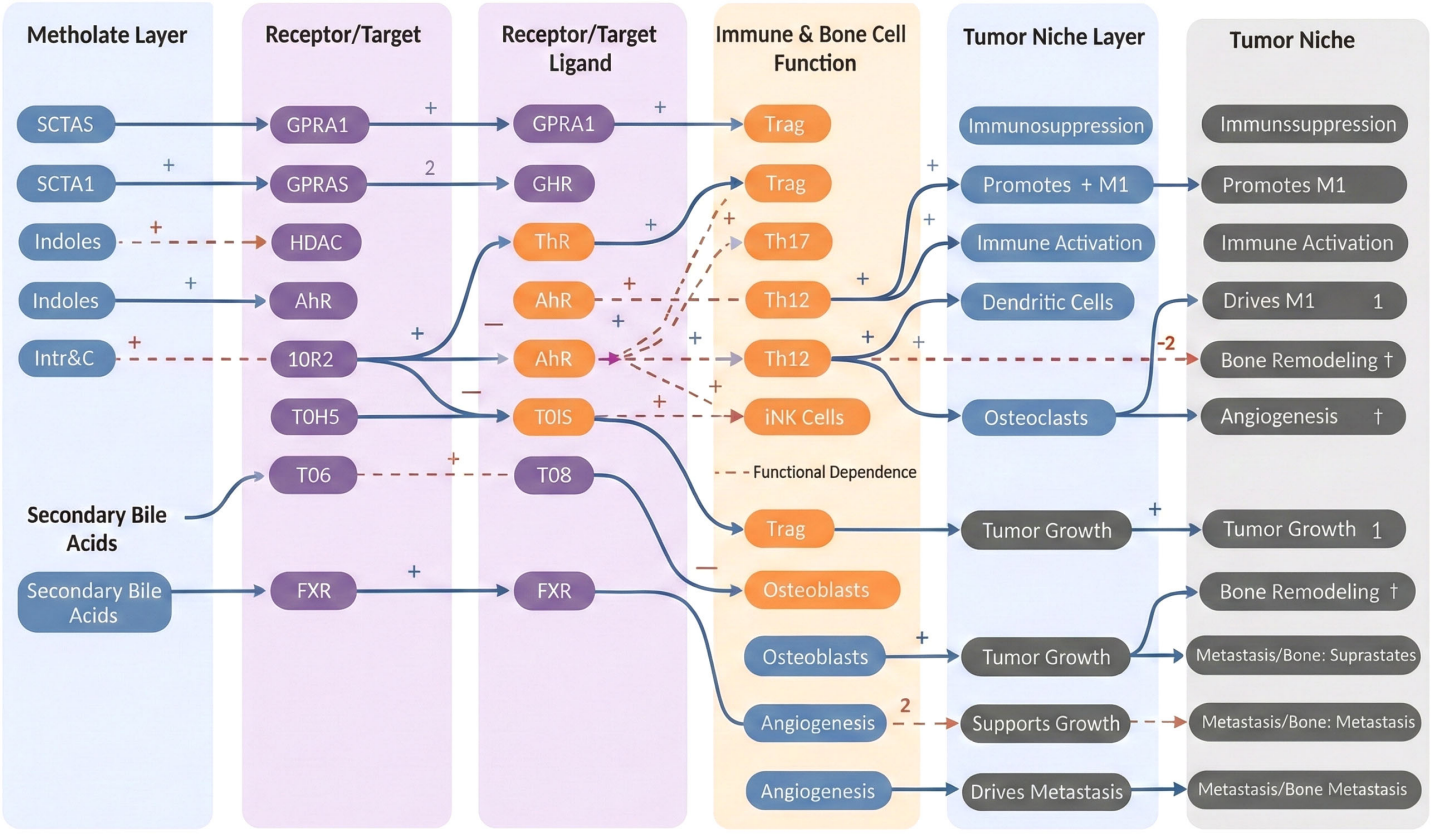
Layered representation of microbiota-derived metabolites, their cognate receptors and downstream immune and bone cell functions that converge on the tumor–bone niche, illustrating how specific metabolite–receptor axes shape immunosuppression, bone remodeling, angiogenesis, tumor growth and bone metastasis.

Building on this cellular and functional framework of the microbiota–immune–bone axis, it is critical to examine metabolite–receptor interactions in greater depth to pinpoint druggable signaling nodes. Accordingly, we systematically integrate and stratify key microbiota-derived metabolites with their cognate host receptors, linking these pairings to downstream effects on immune and bone cell populations and to remodeling of the tumor–bone niche, as summarized in **Figure 3**.

**Figure 3** presents a left-to-right hierarchical map of the signaling cascade by which gut microbiota–derived metabolites, acting through their cognate receptors and molecular targets, reprogram immune and bone cell functions and ultimately shape the tumor-associated bone niche. On the far left, the Metabolite Layer groups representative metabolite classes, including short-chain fatty acids (SCFAs), indole/tryptophan derivatives, and secondary bile acids. Moving rightward, the Receptor/Target and Receptor/Target Ligand layers link these metabolites to corresponding host receptors and targets expressed on immune cells, bone cells, and tumor/stromal cells, including G-protein–coupled receptors (GPR family), the aryl hydrocarbon receptor (AhR), histone deacetylases (HDACs), Toll-like receptors (TLRs), and the bile acid receptor FXR. Experimentally validated activating or inhibitory relationships are denoted by blue solid connectors with “+”/”–” symbols, whereas red dashed connectors indicate putative, context-dependent interactions supported by limited evidence.

Centrally, the Immune & Bone Cell Function layer integrates receptor-mediated inputs and channels them into downstream effector programs. Specific metabolite–receptor pairings can differentially amplify or restrain key T-cell populations—including Treg, Th17, Th1/Th2, and iNKT cells—while concurrently regulating osteoclastogenesis and osteoblast activity, as well as angiogenic processes. Through these coupled immunologic and bone-remodeling effects, the bone marrow microenvironment is dynamically remodeled.

Further right, the Tumor Niche layer consolidates how these cellular outputs translate to tumor-ecosystem behavior, including shifts toward immunosuppression versus immune activation, altered dendritic cell function, osteoclast recruitment, and direct support of tumor proliferation. The terminal Tumor Niche layer summarizes these changes as clinically and pathologically observable outcomes, such as an immunosuppressive microenvironment, skewed M1/M2 macrophage polarization, intensified bone remodeling imbalance, enhanced angiogenesis, accelerated tumor growth, and the emergence of osteolytic, osteoblastic, or mixed metastatic lesions.

Collectively, **Figure 3** underscores multiple druggable intervention points along the continuum of metabolite → receptor/target → immune/bone cell response → tumor-associated bone niche. These relationships suggest that precisely modulating selected microbial metabolites or their receptor-linked signaling pathways may provide a rational strategy to reprogram the tumor-associated bone microenvironment for therapeutic benefit.

**Figure 4:**
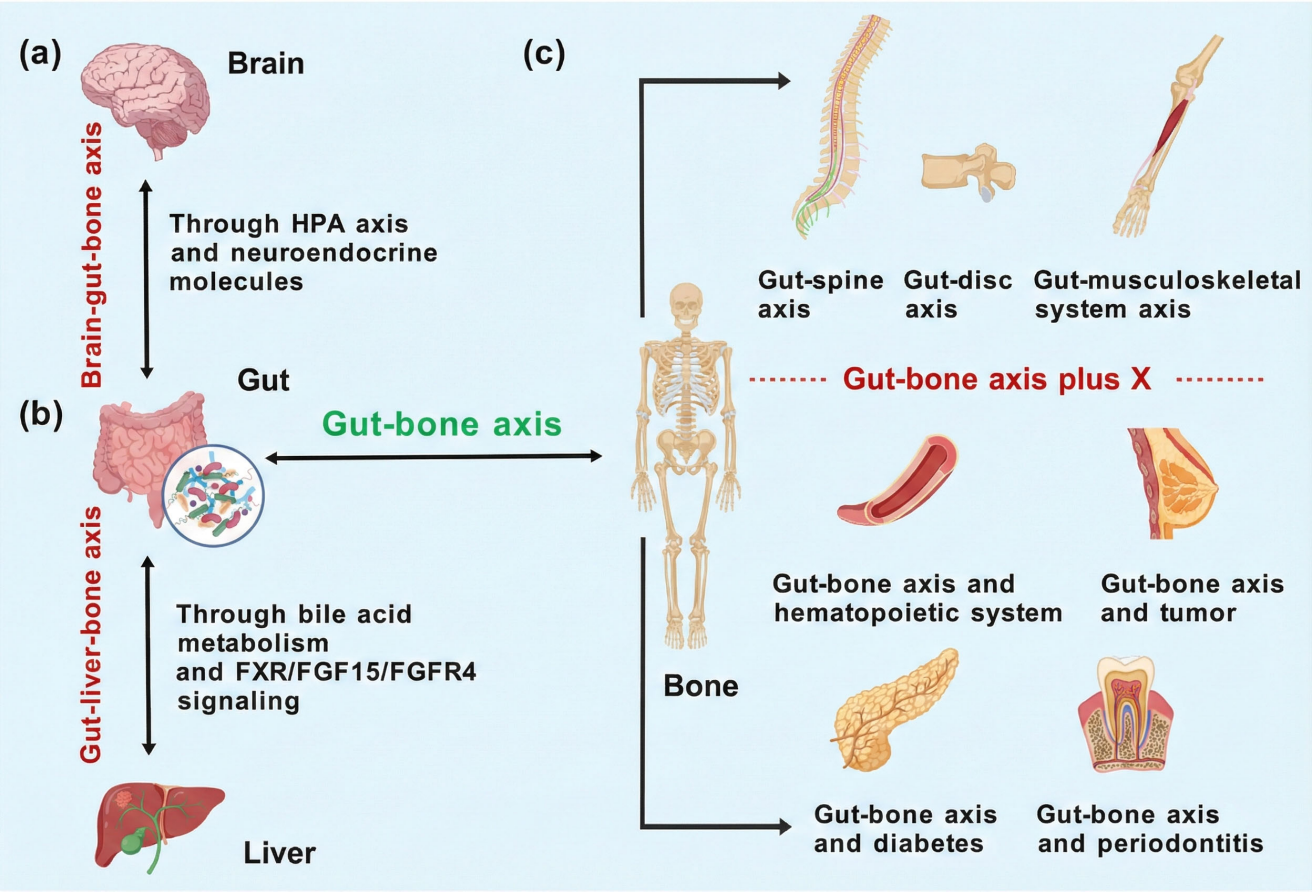
Schematic illustration of the gut–bone axis and its interactions with multiple organ systems. **(a)** The brain–gut axis, mediated through the hypothalamic–pituitary–adrenal (HPA) axis and neuroendocrine molecules, enables bidirectional communication between the brain and the gut. **(b)** The gut–liver axis, in which gut microbiota influence liver function via bile acid metabolism and FXR/FGF15/FGFR4 signaling, contributes to the regulation of the gut–bone axis.**(c)** The gut–bone axis and the expanded “gut–bone axis plus X” concept, highlighting the interactions between the gut and the skeletal system and their links to the spine, intervertebral discs, musculoskeletal system, hematopoietic system, tumors, diabetes, and periodontitis, underscoring the central role of the gut–bone axis in systemic homeostasis and disease.

**Figure 4** extends the gut–bone axis framework by depicting the complex, bidirectional crosstalk between the gastrointestinal tract and the skeletal system, emphasizing cross-organ regulatory networks that collectively govern bone health. By integrating additional systems—including the brain, liver, and musculoskeletal compartments such as the spine, intervertebral discs, and skeletal muscle—the figure highlights the multidimensional routes through which gut-derived cues influence skeletal physiology.

On the left, the brain–gut–bone axis illustrates how hypothalamic–pituitary–adrenal (HPA) axis activity and neuroendocrine mediators shape bone remodeling dynamics. The gut–liver–bone axis focuses on bile acid biotransformation and downstream FXR/FGF15 signaling as a key metabolic–endocrine circuit linking the intestine and liver to skeletal turnover and hepatic function. The gut–bone axis itself is presented not only as a regulator of skeletal homeostasis, but also as a determinant of bone marrow immune-cell composition and activity, thereby coupling metabolite-driven immune modulation to osteogenesis and bone resorption.

Moreover, **Figure 4** highlights several extended axes connecting the gut to the hematopoietic system, diabetes, cancer, and periodontal disease, reinforcing the central influence of the gut microbiota across diverse physiological and pathological contexts. Together, these networks illustrate how microbial dysbiosis can propagate across organ systems, altering immune, metabolic, and inflammatory set points and contributing to systemic disease phenotypes.

Collectively, **Figure 4** frames the gut–bone axis as a multidimensional communication network that helps shape complex conditions—including bone tumors, diabetes, and periodontal disease—while also revealing potential multisystem therapeutic entry points. This expanded perspective provides a conceptual basis for future interdisciplinary investigation and for developing integrative, system-level clinical intervention strategies.

**Figure 5:**
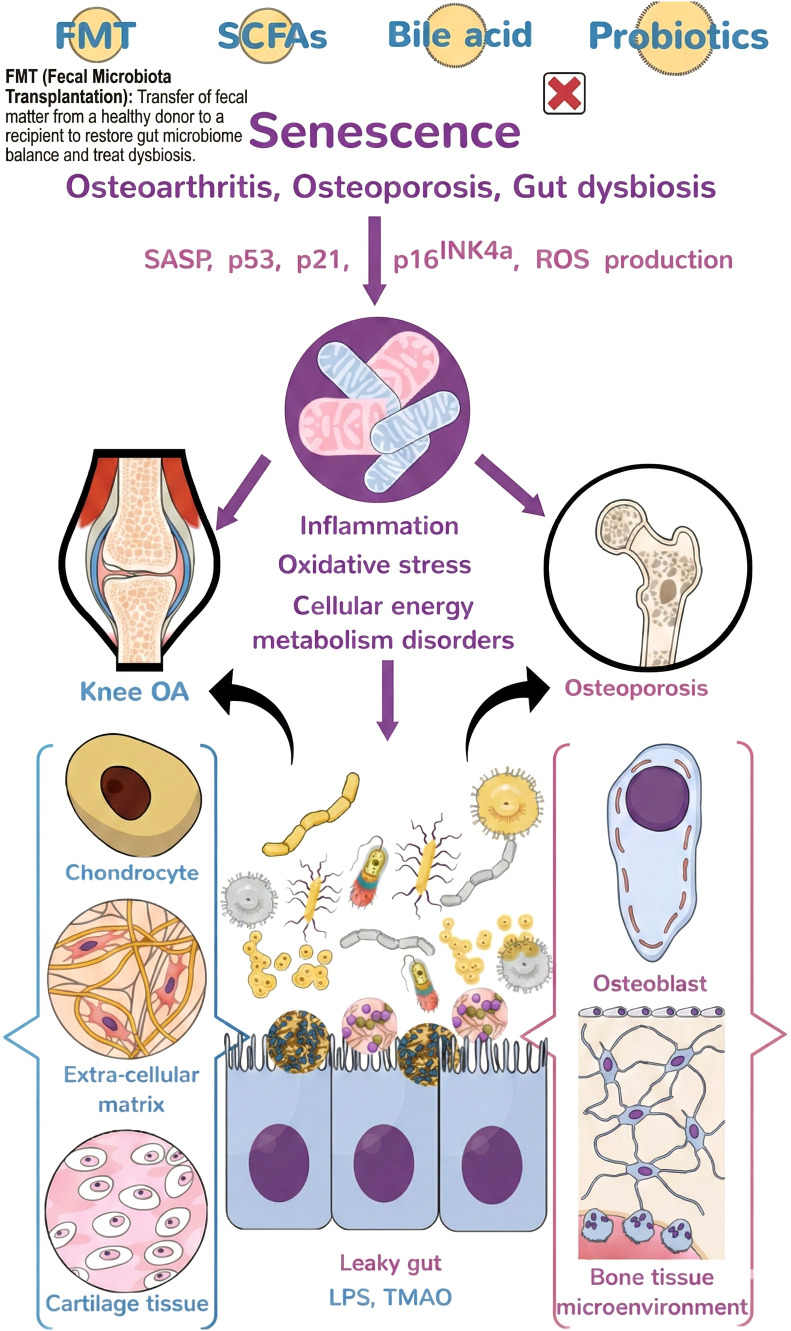
Role of gut microbiota–induced cellular senescence in osteoarthritis and osteoporosis. Gut dysbiosis drives cellular senescence via SASP, p53/p21/p16^INK4a signaling and ROS production, leading to inflammation, oxidative stress, and metabolic dysfunction. These changes promote cartilage degeneration in osteoarthritis and impaired bone remodeling in osteoporosis, while microbiota-targeted interventions (FMT, SCFAs, bile acids, and probiotics) may alleviate these effects.

Building upon the role of the gut microbiota–immune–bone axis in reshaping the bone tumor microenvironment, the same signaling network also participates extensively in the bidirectional coupling between aging-related musculoskeletal disorders and gut microbial dysbiosis. Cellular senescence emerges as a central regulatory hub within this shared pathogenic landscape, as illustrated in **Figure 5**.

This schematic places cellular senescence at the core and integrates how disturbances in the gut microbial ecosystem aggravate the pathological progression of knee osteoarthritis (OA) and osteoporosis through both the *gut–joint axis* and the *gut–bone axis*. At the top of the figure, icons representing FMT, SCFAs, bile acids, and probiotics highlight microbiota- and metabolite-targeted intervention strategies. The central “Senescence” module situates osteoarthritis, osteoporosis, and gut dysbiosis within a unified aging context, marking classical hallmarks of senescence—including SASP, p53, p21, p16^INK4a, and ROS generation. Arrows radiating outward from this central hub lead to knee OA on the left and osteoporosis on the right, underscoring the roles of inflammaging, oxidative stress, and cellular energy dysfunction in driving cartilage degradation, subchondral bone alterations, impaired osteoblast function, and enhanced osteoclastogenesis.

## Discussion

The expanding understanding of the microbiota–immune–bone (MIB) axis provides a conceptual and mechanistic bridge connecting intestinal microbial ecology to bone remodeling dynamics and bone tumor biology (Choy et al., 2017). However, despite rapid gains in foundational knowledge, key gaps remain. First, the causal hierarchy linking microbial dysbiosis to tumor-permissive bone remodeling has not been firmly established. Much of the current evidence derives from cross-sectional profiling, correlative microbial “signatures,” or reductionist systems that isolate single metabolites or individual immune subsets. These fragmented paradigms limit our ability to model the integrated, emergent behavior of the full microbiota–immune–bone (MIB) axis. Second, the best-characterized microbial cues to date—including SCFAs, bile acids, and indole derivatives—often display context-dependent and even bidirectional effects across tissues and disease states, which complicates mechanistic attribution and therapeutic translation. For example, SCFAs can promote Treg differentiation and suppress inflammation under physiological conditions, yet in certain tumor settings they may enhance immune tolerance, inadvertently facilitating tumor escape. Similarly, bile-acid receptor signaling through FXR/TGR5 exerts divergent outcomes depending on tissue localization, metabolic state, and crosstalk with endocrine pathways (Fiorucci et al., 2009; Baars et al., 2015; Perino et al., 2021).

Neuroendocrine regulation represents a critical but often under integrated component of the microbiota–immune–bone axis. Gut microbiota–dependent modulation of neuroendocrine factors, including intestinally derived serotonin and hypothalamic–pituitary–adrenal axis signaling, exerts coordinated effects on both immune-cell function and bone remodeling. These neuroendocrine cues influence immune-cell polarization, inflammatory tone, and osteoclast–osteoblast coupling, thereby shaping the permissiveness of the bone tumor microenvironment. Importantly, neuroendocrine regulation does not act in isolation but functions as an integrative layer that translates microbial signals into immune and skeletal responses relevant to tumor progression.

Another challenge lies in the heterogeneity of bone tumors and bone metastases, whose metabolic dependencies, stromal interactions, and immune landscapes differ drastically across tumor types (Clézardin et al., 2021; Hofbauer et al., 2021). Consequently, an identical microbial shift—or even the same immune perturbation—may yield divergent skeletal outcomes across bone malignancies such as osteosarcoma, multiple myeloma, and metastatic prostate cancer. In addition, the ongoing interplay between osteoclast-driven bone resorption and osteoblast-driven bone formation generates spatially heterogeneous metastatic niches, producing microhabitats that can differentially support distinct tumor subclones. Integrating this spatial and ecological complexity into MIB-axis investigations therefore remains a major unmet need.

Despite the rapidly expanding literature linking gut microbiota, immunity, and bone biology, important limitations remain. Most studies adopt reductionist approaches focusing on individual microbial taxa, metabolites, or immune-cell subsets, which limits their ability to capture the dynamic and context-dependent interactions governing bone remodeling and tumor progression. Critically, such fragmented models fail to explain divergent skeletal outcomes across tumor types and disease stages, underscoring the need for an integrative, systems-level framework such as the microbiota–immune–bone (MIB) axis.

Beyond the direct influence on the bone microenvironment, the MIB axis may also play a critical role in modulating tumor cell dissemination and their ability to overcome various physiological barriers during metastasis. For instance, the formidable blood-brain barrier (BBB) presents a significant challenge for tumor cells metastasizing to the central nervous system. While our review primarily focuses on bone metastasis, the general principles of how gut microbiota and their metabolites can influence systemic immunity and vascular integrity are broadly relevant to tumor cell extravasation across diverse anatomical sites. Dysbiosis-driven inflammation or specific microbial metabolites (such as short-chain fatty acids and bile acids) can modulate endothelial cell permeability and activate immune cells, which in turn may facilitate or impede tumor cell transit across vascular walls into secondary organs. Investigating how the MIB axis specifically modulates the integrity and permeability of specialized organ barriers, such as the BBB or the distinct sinusoidal architecture within the bone marrow, represents a crucial frontier for understanding metastatic tropism and developing novel therapeutic strategies that target these intricate interactions.

Current preclinical findings are not always directly translatable to clinical practice. Differences between mouse and human microbiota, as well as the influence of diet, antibiotics, geographical environment, and host genetics, introduce confounders that complicate interpretation (Nguyen et al., 2015; Rothschild et al., 2018; Parizadeh and Arrieta, 2023). Standardization of microbiome research methodologies, coupled with longitudinal human cohort studies, will be essential for identifying robust, clinically actionable microbial biomarkers and therapeutic targets (Shukla et al., 2024; Joos et al., 2025).

## Conclusion

The microbiota–immune–bone (MIB) axis represents a dynamic, multilevel regulatory network through which the gut microbial ecosystem shapes bone remodeling and tumor evolution. By integrating microbiota-derived metabolites, receptor-mediated immune reprogramming, osteoclast–osteoblast coupling, and tumor–stromal crosstalk, the MIB axis provides a unifying framework for understanding bone tumor initiation, progression, and treatment response. Accumulating evidence further points to multiple druggable checkpoints along this continuum, creating opportunities for upstream therapeutic intervention. Despite persistent challenges—including mechanistic nonlinearity, inter-individual heterogeneity, and translational constraints—recent conceptual and technological advances provide a strong foundation for microbiota-informed strategies. Ultimately, rational targeting of the MIB axis may enable reconditioning of the tumor-associated bone microenvironment, enhance therapeutic efficacy, and advance precision medicine for patients with bone malignancies.
